# Circulating levels of ATP is a biomarker of HIV cognitive impairment

**DOI:** 10.1016/j.ebiom.2019.10.029

**Published:** 2019-12-03

**Authors:** Stephani Velasquez, Lisa Prevedel, Silvana Valdebenito, Anna Maria Gorska, Mikhail Golovko, Nabab Khan, Jonathan Geiger, Eliseo A. Eugenin

**Affiliations:** a Department of Neuroscience, Cell Biology, and Anatomy, University of Texas Medical Branch (UTMB), Research Building 17, Fifth Floor, 105 11th Street, Galveston, Texas, 77555, USA; b Department of Biomedical Sciences, University of North Dakota School of Medicine and Health Sciences, Grand Forks, North Dakota, USA

**Keywords:** Anti-retroviral/dementia/HIV-1 reservoirs/NeuroHIV/Pannexin

## Abstract

**Background:**

In developed countries, Human Immunodeficiency Virus type-1 (HIV-1) infection has become a chronic disease despite the positive effects of anti-retroviral therapies (ART), but still at least half of the HIV infected population shown signs of cognitive impairment. Therefore, biomarkers of HIV cognitive decline are urgently needed.

**Methods:**

We analyze the opening of one of the larger channels expressed by humans, pannexin-1 (Panx-1) channels, in the uninfected and HIV infected population (*n* = 175). We determined channel opening and secretion of intracellular second messengers released through the channel such as PGE_2_ and ATP. Also, we correlated the opening of Panx-1 channels with the circulating levels of PGE_2_ and ATP as well as cogntive status of the individuals analyzed.

**Findings:**

Here, we demonstrate that Panx-1 channels on fresh PBMCs obtained from uninfected individuals are closed and no significant amounts of PGE_2_ and ATP are detected in the circulation. In contrast, in all HIV-infected individuals analyzed, even the ones under effective ART, a spontaneous opening of Panx-1 channels and increased circulating levels of PGE_2_ and ATP were detected. Circulating levels of ATP were correlated with cognitive decline in the HIV-infected population supporting that ATP is a biomarker of cognitive disease in the HIV-infected population.

**Interpretation:**

We propose that circulating levels of ATP could predict CNS compromise and lead to the breakthroughs necessary to detect and prevent brain compromise in the HIV-infected population.

## Research in context

### Evidence before this study

Currently, there are no biomarkers to predict or identify brain compromise in the HIV-infected population. HIV-associated neurocognitive disorders (HAND) occurs in at least 50% of the HIV-infected population despite effective ART. Commonly, CNS damage in the HIV-infected population is assessed by neurophysiological testing batteries and by imaging techniques. Several groups had proposed and examined potential biomarkers of brain disease, such as neurofilament (NFL). NFL is released into the CSF as a result of neuronal damage. Though NFL is a late representation of brain damage and it is not specific for HIV. In addition, CSF is difficult and painful to obtain. Thus, the necessity for a peripheral and/or central nervous system (CNS) biomarker of cognitive compromise in the HIV-infected population is urgent.

### Added-value of this study

Our data identify the mechanism by which a potential biomarker of CNS disease, ATP, is released into the circulation and the impact of this biomarker in blood-brain barrier function, stability, and neuroinflammation. ATP levels in serum were predictive of CNS damage and cognitive decline in the HIV-infected population. We identified that circulating levels of ATP can be used as an early biomarker of HIV-brain compromise.

### Implications of all the available evidence

Overall, our results indicate that circulating levels of ATP is a useful biomarker of cognitive disease in the HIV-infected population. Therefore, we propose that regular determination of circulating levels of ATP could identify individuals under risk of cognitive disease. Furthermore, blocking Panx-1 channels or activation of purinergic receptors can generate clinical interventions to prevent CNS damage in the HIV-infected population.

## 1 Introduction

The pathogenesis of HIV-infection involves a series of dynamic interactions between HIV and several host proteins to support effective HIV-infection, replication, generation of viral reservoirs, and associated inflammation [1], [2], [3]. Despite effective antiretroviral therapy (ART), most HIV-infected individuals still showed strong evidence of chronic systemic and brain inflammation resulting in cognitive impairment. However, the mechanisms of CNS damage are still elusive.

Recently, we and others identified a novel host protein involved in HIV entry and replication, named pannexin-1 (Panx-1) [4], [5], [6], [7], [8], [9]. Pannexin-1 proteins form a large plasma membrane channel that, upon opening, allows the release of several intracellular mediators, including ATP and other nucleotides, prostaglandins, glutamate, NAD^+,^ and metabolites such as glucose. In physiological conditions, these channels remain closed. However, in pathological conditions, including HIV-infection, these channels open to amplify inflammation and HIV infection/latency/reactivation [8], [10], [11], [12]. In the context of HIV, we and others identified that the binding of HIV to CD4 receptor and CXCR4 and/or CCR5 co-receptors, induces opening of Panx-1 channels, resulting in local ATP release through the pore and subsequent autocrine and paracrine purinergic receptor activation, which accelerates HIV entry into immune cells [12,13].

Our current study demonstrated: first, Panx-1 channels are closed in PBMCs isolated from uninfected individuals, with low to undetectable circulating levels of ATP and PGE_2_ as expected; second, fresh PBMCs isolated from HIV-infected individuals have spontaneous opening of Panx-1 channels, despite the fact that most of the individuals analyzed had low to undetectable HIV-replication and normal CD4 counts; third, all HIV-infected individuals analyzed had increased circulating levels of PGE_2_ and ATP, both inflammatory compounds released through the opening of Panx-1 channels; and fourth, circulating ATP levels, but not PGE_2_, is a biomarker of HIV cognitive disease. Further, we identified that the concentrations of ATP associated with cognitive impairment resulted in compromise of the blood-brain barrier (BBB) and increase transmigration of leukocytes across the BBB, a critical hallmark of NeuroHIV, in a Panx-1 dependent manner. Overall, our results indicate that circulating levels of ATP are a useful biomarker of cognitive disease in the HIV-infected population. Therefore, we propose that blocking Panx-1 channels or targeting the circulating ATP can provide an excellent therapeutic intervention in all HIV-infected individuals at risk of cognitive disease.

## 2 Materials and methods

Subjects**.** Serum/plasma samples were obtained from the National NeuroAIDS Tissue Consortium (NNTC, www.nntc.org) and the CNS HIV Antiretroviral Therapy Effects Research (CHARTER) *n* = 175. All samples had a cognitive assessment and other clinical information available, as described in Table 1. All the analyses were performed blindly.

**Table 1:** Patient Information.

Patient number	HIV status	Age	Gender	Cognitive assessment	CD4 counts(cells/mm)	Viral Load(copies/ml)	Years with HIV
1	+	30	M	HAD	371	21,085	21
2	+	30	M	MND	185	2544	16
3	+	31	M	N.I.	129	40	19
4	+	31	M	N.I.	219	U.D.	21
5	+	31	M	N.I.	108	50	24
6	+	31	M	MND	93	1988	24
7	+	32	M	MND	70	1054	N.R.
8	+	32	M	MND	18	8645	N.R.
9	+	32	M	MND	77	5365	24
10	+	42	M	MND	12	1005	24
11	+	42	M	HAD	2	10,545	24
12	+	42	M	HAD	25	105,264	9
13	+	43	M	MND	31	20,344	9
14	+	43	M	HAD	3	10,256	11
15	+	43	M	HAD	7	8685	15
16	+	39	M	MND	11	2015	16
17	+	39	M	HAD	4	81,975	4
18	+	39	M	HAD	9	26,759	18
19	+	39	F	MND	5	62,357	19
20	+	39	M	HAD	8	20,157	7
21	+	40	M	HAD	4	201,545	7
22	+	40	F	HAD	5	156,841	6
23	+	43	M	MND	21	27,951	13
24	+	38	M	N.I.	185	3678	11
25	+	38	M	MND	64	2015	7
26	+	39	M	MND	70	68,951	12
27	+	39	M	HAD	70	48,001	15
28	+	40	M	N.I.	81	2687	10
29	+	41	M	MND	74	2468	9
30	+	42	M	N.I.	92	50	17
31	+	42	M	MND	197	60,257	N.R.
32	+	43	M	HAD	86	298,564	15
33	+	43	F	HAD	66	128,978	15
34	+	34	M	MND	48	50,004	19
35	+	35	M	MND	40	63,904	7
36	+	33	M	HAD	9	3045	6
37	+	52	M	N.I.	172	50	6
38	+	43	F	N.I.	112	50	9
39	+	45	M	MND	499	<20	14
40	+	50	M	MND	334	<20	15
41	+	47	F	N.I.	365	169	10
42	+	52	F	N.I.	253	2005	3
43	+	42	F	N.I.	630	<20	5
44	+	40	F	MND	473	U.D.	7
45	+	52	F	MND	299	U.D.	12
46	+	49	F	N.I.	440	50	12
47	+	42	F	N.I.	241	50	12
48	+	62	F	MND	224	<20	18
49	+	42	F	MND	105	50	14
50	+	37	F	MND	312	<20	15
51	+	69	F	MND	518	<20	13
52	+	37	F	N.I.	231	168	8
53	+	53	M	N.I.	199	865	21
54	+	57	M	N.I.	409	U.D.	19
55	+	66	M	MND	422	<20	19
56	+	36	M	N.I.	510	U.D.	10
57	+	78	F	N.I.	436	<20	19
58	+	71	F	N.I.	560	U.D.	18
59	+	62	F	N.I.	358	265	17
60	+	35	M	N.I.	300	1002	14
61	+	52	M	N.I.	299	2451	9
62	+	36	M	N.I.	356	U.D.	9
63	+	56	M	MND	409	1024	8
64	+	52	M	N.I.	504	U.D.	24
65	+	83	M	N.I.	425	U.D.	24
66	+	52	F	N.I.	468	U.D.	21
67	+	45	F	MND	278	13,645	19
68	+	45	M	N.I.	308	<20	18
69	+	43	M	MND	109	199	18
70	+	52	M	N.I.	323	U.D.	13
71	+	58	M	N.I.	171	U.D.	27
72	+	48	F	MND	199	20	15
73	+	43	F	HAD	401	81	17
74	+	43	M	N.I.	392	U.D.	21
75	+	32	F	MND	185	201	17
76	+	27	M	MND	225	99	13
77	+	29	F	N.I.	407	U.D.	12
78	+	41	M	MND	399	N.R.	8
79	+	41	F	N.I.	510	20	5
80	+	58	M	NPI-O	427	57	7
81	+	51	M	N.I.	327	80	9
82	+	33	F	N.I.	317	U.D.	13
83	+	60	F	HAD	300	U.D.	19
84	+	62	M	MND	280	U.D.	22
85	+	65	M	N.I.	880	34	27
86	+	58	F	N.I.	45	692	31
87	+	63	M	N.I.	951	<20	28
88	+	73	M	MND	253	<20	22
89	+	70	M	N.I.	422	<20	24
90	+	63	M	N.I.	891	U.D.	15
91	+	60	F	N.I.	264	U.D.	14
92	+	61	F	MND	1126	U.D.	29
93	+	71	M	N.I.	892	U.D.	18
94	+	74	M	N.I.	680	U.D.	23
95	+	66	F	HAD	681	24	19
96	+	51	M	NPI-O	1053	82	17
97	+	49	F	HAD	518	2776	14
98	+	67	F	NPI-O	480	5493	24
99	+	43	F	HAD	83	19,531	14
100	+	48	M	NPI-O	51	57,880	17
101	+	50	F	HAD	451	20	20
102	+	42	F	HAD	1422	U.D.	22
103	+	62	F	N.I.	376	U.D.	21
104	+	50	M	MND	1254	U.D.	14
105	+	49	F	MND	761	25	23
106	+	61	F	MND	488	34	24
107	+	50	M	NPI-O	271	134	11
108	+	55	F	N.I.	415	20	27
109	+	70	M	N.I.	544	U.D.	26
110	+	61	M	MND	353	U.D.	27
111	+	48	F	NPI-O	731	U.D.	3
112	+	54	F	HAD	197	U.D.	29
113	+	55	F	MND	574	U.D.	24
114	+	49	F	NPI-O	823	U.D.	3
115	+	65	F	MND	604	U.D.	12
116	–	48	F	N.D.	N.D.	N.A.	N.A.
117	–	32	F	N.D.	N.D.	N.A.	N.A.
118	–	45	M	N.D.	N.D.	N.A.	N.A.
119	–	47	F	N.D.	N.D.	N.D.	N.D.
120	–	51	M	N.D.	N.D.	N.D.	N.D.
121	–	68	M	N.D.	N.D.	N.D.	N.D.
122	–	38	M	N.D.	N.D.	N.A.	N.A.
123	–	38	M	N.D.	N.D.	N.A.	N.A.
124	–	42	F	N.D.	N.D.	N.A.	N.A.
125	–	44	F	N.D.	N.D.	N.A.	N.A.
126	–	49	M	NPI-O	N.D.	N.A.	N.A.
127	–	51	F	N.D.	N.D.	N.A.	N.A.
128	–	58	F	N.D.	N.D.	N.A.	N.A.
129	–	61	F	N.D.	N.D.	N.A.	N.A.
130	–	67	M	N.D.	N.D.	N.A.	N.A.
131	–	27	F	NPI-O	N.D.	N.A.	N.A.
132	–	26	F	N.D.	N.D.	N.A.	N.A.
133	–	31	M	N.D.	N.D.	N.A.	N.A.
134	–	34	M	N.D.	N.D.	N.A.	N.A.
135	–	39	M	N.D.	N.D.	N.A.	N.A.
136	–	45	F	NPI-O	N.D	N.A.	N.A.
137	–	62	F	N.D.	N.D.	N.A.	N.A.
138	–	53	M	N.D.	N.D.	N.A.	N.A.
139	–	39	F	N.D.	N.D.	N.A.	N.A.
140	–	39	M	N.D.	N.D.	N.A.	N.A.
141	–	40	M	N.D.	N.D.	N.A.	N.A.
142	–	47	M	N.D.	N.D.	N.A.	N.A.
143	–	49	F	N.D.	N.D.	N.A.	N.A.
144	–	52	M	N.D.	N.D.	N.A.	N.A.
145	–	58	M	N.D.	N.D.	N.A.	N.A.
146	–	61	M	N.D.	N.D.	N.A.	N.A.
147	–	62	M	N.D.	N.D.	N.A.	N.A.
148	–	33	M	N.D.	N.D.	N.A.	N.A.
149	–	39	F	N.D.	N.D.	N.A.	N.A.
150	–	39	M	NPI-O	N.D.	N.A.	N.A.
151	–	40	M	N.D.	N.D.	N.A.	N.A.
152	–	35	M	N.D.	N.D.	N.A.	N.A.
153	–	39	F	N.D.	N.D.	N.A.	N.A.
154	–	42	F	N.D.	N.D.	N.A.	N.A.
155	–	35	M	N.D.	N.D.	N.A.	N.A.
156	–	52	M	N.D.	N.D.	N.A.	N.A.
157	–	55	M	N.D.	N.D.	N.A.	N.A.
158	–	43	M	N.D.	N.D.	N.A.	N.A.
159	–	45	F	N.D.	N.D.	N.A.	N.A.
160	–	30	F	N.D.	N.D.	N.A.	N.A.
161	–	31	M	N.D.	N.D.	N.A.	N.A.
162	–	42	M	NPI-O	N.D.	N.A.	N.A.
163	–	39	M	N.D.	N.D.	N.A.	N.A.
164	–	43	M	N.D.	N.D.	N.A.	N.A.
165	–	45	M	N.D.	N.D.	N.A.	N.A.
166	–	38	M	N.D.	N.D.	N.A.	N.A.
167	–	39	M	N.D.	N.D.	N.A.	N.A.
168	–	38	M	N.D.	N.D.	N.A.	N.A.
169	–	43	M	N.D.	N.D.	N.A.	N.A.
170	–	43	F	N.D.	N.D.	N.A.	N.A.
171	–	47	F	N.D.	N.D.	N.A.	N.A.
172	–	52	F	N.D.	N.D.	N.A.	N.A.
173	–	56	M	N.D.	N.D.	N.A.	N.A.
174	–	55	F	N.D.	N.D.	N.A.	N.A.
175	–	57	M	N.D.	N.D.	N.A.	N.A.

Notes: M: male; F: Female; N.A.: not applicable; N.D.: not determined; N.R.: not recorded. NI: No impairment; MND: Mild Neurocognitive Disorder; HAD: HIV associated dementia; NPI-O: Neurocognitive impairment other or UD: undetectable.

Neurocognitive examination. NNTC and CHARTER perform a comprehensive neurocognitive test battery every 6 months, including motor function (perceptual-motor speed), verbal fluency, executive function, attention/working memory, speed of information processing, learning, and memory [14], [15], [16], for details, see http://www.mountsinai.org/patient-care/service-areas/neurology/areas-of-care/neuroaids-program or https://nntc.org/.

Fresh blood from local participants**.** For live-cell imaging experiments, 10 to 15 mL of blood from HIV-seropositive participants were obtained through MHBB, a research resource operating at the Icahn School of Medicine at Mount Sinai (New York, NY), from uninfected volunteers at Rutgers University (Newark, NJ), or from leukopacks from the NY/NJ Blood Center. HIV-positive individuals were assayed for CD4 cell counts, plasma viral loads and urine toxicology, and underwent neuropsychological testing at the time of blood draw. Patient demographic and virological information is listed in Table 1. Patients gave written, informed consent for the provision of blood for the purposes of HIV research before inclusion in the current CHARTER pilot study. The protocol for blood collection and analysis was approved by the Mount Sinai, Rutgers University and University of Texas Medical Branch (UTMB) Institutional Review Board (Protocol Numbers, Pro20140000794, Pro2012001303, 18–0136, 18–0135, 18–0134).

Isolation of human PBMCs and CD4^+^ *T* lymphocytes**.** After removing the plasma, PBMCs were isolated by over-layering with Ficoll-Paque (Cat# GE17-1440–02, Amersham Bioscience, Uppsala, Sweden) according to the procedure described by the manufacturer. PBMCs were isolated within 4 h of blood draw. All described analysis was performed on freshly isolated blood.

Dye uptake and time-lapse microscopy**.** To characterize the functional state of Panx-1 channels, dye-uptake experiments using ethidium (Etd) bromide were performed (Cat # 15,585,011, ThermoFisher, Grand Island, NY, USA). Cells were washed twice in HBSS and then exposed to Locke's solution (containing 154 mM NaCl, 5.4 mM KCl, 2.3 mM CaCl_2_, 5 mM HEPES, and pH 7.4) with 5 μM Etd and time-lapse microscopy were then performed. Phase-contrast and fluorescence microscopy with time-lapse imaging were used to record cell appearance and fluorescence-intensity changes in each condition. Fluorescence was recorded every 30 s. The NIH ImageJ program was used for off-line image analysis and fluorescence quantification. For data representation and calculation of Etd uptake slopes, the average of two independent background fluorescence (FB) (expressed as A.U.) was subtracted from mean fluorescent intensity (F1). Results of this calculation (F1−FB), for at least 20 cells, were averaged and plotted against time (expressed in minutes). Slopes were calculated using Microsoft Excel software and expressed as A.U./min. The microscope and camera settings remained the same in all experiments. Dead cells or cells with a damaged plasma membrane were identified during the time-lapse microscopy as a result of their nonspecific Etd uptake rate, determined by lack of time dependency and stability in dye uptake (not inhibited by channel blockers), and were not quantified.

ATP Assay**.** Plasma/serum was collected before PBMC separation, and ATP concentration was determined using the ATPlite luminescence assay system (PerkinElmer, MA) by combining 100 μL of the sample with 100 μL of ATPlite reagent. Luminescence was measured using a PerkinElmer EnVision Multilabel Plate Reader. The extracellular concentration of ATP was determined by comparing sample luminescence to a standard curve generated using ATP standards provided by the manufacturer. To assure rigor in our determinations, some samples were submitted for blinded analysis of ATP levels using mass spectrometry (University of North Dakota, ND).

Analysis of IL-1β and PGE_2_ release**.** Plasma/serum was collected, divided into aliquots, and stored at −80ºC. There were no freeze-thaw cycles before analysis. Plasma/serum was analyzed for TNF-α, IL-1β (Quantikine ELISA kit; R and D Systems, Minneapolis MN, USA) and PGE_2_ (Abcam, Cambridge, MA, USA) by enzyme-linked immunosorbent assay (ELISA) according to the manufacturer's instructions.

Blood-brain barrier (BBB) model. This in vitro BBB model consists of primary human BMVEC and primary human astrocytes in co-culture on opposite sides of a gelatin-coated, 3 µm pore-size tissue culture insert (Falcon, BD, Franklin Lakes, NJ) as we described [17], [18], [19], [20], [21]. Co-cultures were maintained for three days to enable contact between astrocyte endfeet with BMVEC on the opposite side of the model as described [17]. After this, the BBB model was treated with different ATP concentrations (Cat# A1852, Sigma Chemical Co., St. Louis, MO, USA), and BBB permeability was measured using BSA conjugated to Evans Blue, as we described[17].

Transmigration assays of mononuclear cells across the model of the human BBB. Three x 10^5^ PBMCs in M199 culture medium (Cat# 31,100,035, ThermoFisher, Grand Island, NY, USA) with 10% FBS was added to the top of each tissue culture insert as described [17], [22]. After 24 h the number of cells that had transmigrated in response to CCL2 (100 or 500 ng/ml) or without chemoattractant added to the lower chamber was analyzed by FACScan using premixed human CD45 (RRID: AB_10,852,703) and CD14 (RRID: AB_10,598,367) monoclonal antibodies conjugated to FITC and PE, respectively [17], [22].

Statistical analysis**.** Statistical analyses were performed using Prism 5.0 software (GraphPad Software, Inc., San Diego, CA). Analysis of variance was used to compare the different groups; *P ≤ 0.001 for all statistical analyses performed in this study.

## 3 Results

### 3.1 Participant demographics

We collected 175 plasma/serum samples from uninfected (*n* = 60) and HIV-infected individuals (*n* = 115). There were no differences between the HIV-negative and HIV-positive individuals in age (HIV-positive, mean = 47.9 ± 12.2 years; HIV-negative, mean = 45.3 ± 10.1 years; Tables 1 and 2) and gender (HIV-positive = 39% female and 61% male; HIV-negative = 39% female and 61% male; Table 1 and 2). The HIV-positive cohort had an average of 15.9 ± 6.8 years of living with HIV, an average CD4 count of 323.2 ± 285.7 cells/μl, and mean plasma HIV RNA of 14,452 ± 41,779 log copies/ml (range from undetectable to 57,880). Approximately 28% of participants had an undetectable viral load (see Table 1 and 2). Among the HIV-positive participants, 69% had some degree of cognitive impairment, as determined by neuropsychological testing (Table 1).

**Table 2:** Demographic of HIV positive and HIV negative participants, N/*A* = Not Applicable.

	HIV Positive (*n* = 115) Mean ± SD	HIV Negative (*n* = 60) Mean ± SD
Patient Demographics		
Age (Years)	47.89 ± 12.23	45.3 ± 10.12
% Female	39%	39%
% Male	61%	61%
Years w/ HIV	15.87 ± 6.8	N/A
Immunovirologic information		
CD4 T cell count, cells/ μl	323.2 ± 285.7 14,452 ± 41,779	N/AN/A
Plasma HIV RNA, log copies/ml	47%	N/A
% w/ undetectable viral loads	28%	N/A
% w/ cognitive impairment	69%	N/A

To assure an unbiased assessment of the serum/plasma samples, all samples were received and analyzed blindly. Only after all the data was acquired, clinical and HIV status was requested. With regard to HIV-associated CNS disease, our population ranged from no impairment to HIV-associated dementia. Table 2 summarizes the information of each individual analyzed including age, gender, years with HIV, CD4 count, plasma HIV RNA copies, and cognitive status.

### 3.2 Pannexin-1 channels are in a closed state in uninfected individuals

Our previous published data indicate that binding of HIV to CD4 and CCR5/CXCR4 co-receptors results in the transient opening of Panx-1 channels to enable the virus to fuse with the plasma membrane. In contrast, in uninfected samples, the channel remains closed [8,12,23]. However, the chronic effects of HIV-infection on Panx-1 channel activity were unknown. To determine the status of the channel, Panx-1 channel opening was determined by Ethidium (Etd, 5 µM) uptake rate. Ethidium only crosses the plasma membrane in healthy cells by passing through specific large channels, such as Connexin (Cx) and Pannexin (Panx) hemichannels, and its rate of intracellular accumulation is reflective of channel opening and closing [24,25].

**Fig. 1:**
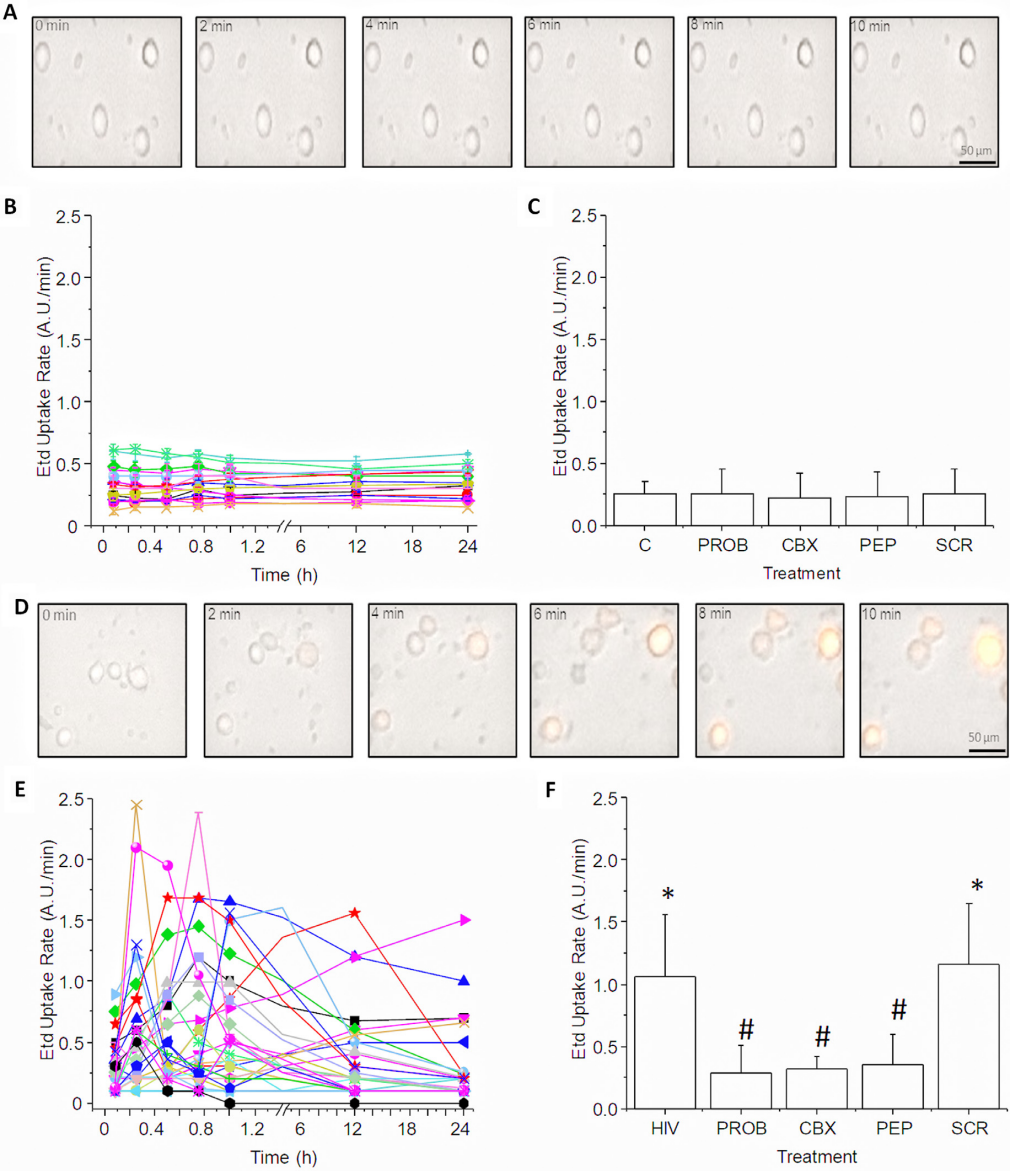
PBMCs isolated from uninfected individuals had closed Panx-1 channels. In contrast, PBMCs isolated from HIV-infected individuals had a spontaneous opening of Panx-1 channels. (A) Uptake of Ethidium (Etd) was quantified by time-lapse microscopy at different time points up to 24 h, the last point assayed. Ethidium can only cross the plasma membrane through hemichannels due to its high molecular weight. Only live cells with increasing uptake of Etd were quantified to discount Etd uptake of dead cells. A representative set of images of live cell imaging recording. (B) Quantification of Etd uptake rate indicates that in all uninfected individuals analyzed, the Panx-1 channel on the surface of PBMCs were in a closed stage. Each line represents data from a single individual. (C) Blockers of Panx-1 channels such as Probenecid (PROB, 500 µM), carbenoxolone (CBX, 50 µM), pannexin-1 blocking peptide (PEP, 300 µM) as well as the negative control, scrambled peptide (SCR) did not show any unspecific Etd uptake. (*n* = 30). (D) As indicated above, uptake of Ethidium (Etd) was quantified by time-lapse microscopy at different time points up to 24 h, the last point assayed. A representative set of images of live cell imaging recording from HIV-infected samples is shown. Fluorescence is accumulated in a time-dependent manner inside of PBMCs. Most of these PBMCs come from HIV-infected cells with low to undetectable viral replication (see table 1). (E) Quantification of Etd uptake rate indicates that Panx-1 channels on the surface of PBMCs were in an open stage in all individuals analyzed (*n* = 60). Each line represents data from a single individual. (F) Blockers of Panx-1 channels such as Probenecid (PROB, 500 µM), carbenoxolone (CBX, 50 µM), or pannexin-1 blocking peptide (PEP, 300 µM) were able to prevent the opening of the Panx-1 channels and Etd uptake observed in HIV-infected conditions confirming the identity of the channel analyzed. Scrambled peptide (SCR) did not show any unspecific effect. (*n* = 60). * Correspond to significance compared to control conditions as observed in Fig. 1, *p ≤ 0.005, *n* = 60; # *p* ≤ 0.003, *n* = 60, as compared to HIV conditions. The data are expressed as mean ± SD.

No significant changes in Etd uptake rate were detected in PBMCs obtained from uninfected individuals (Fig. 1A and 1B, *n* = 45 different individuals). We performed experiments up to 25 h of recording with minimal to no Etd uptake detected (Fig. 1B, each curve represent a different individual analyzed). Pooling all the data from PBMCs obtained from 45 different uninfected individuals indicated a low to undetectable dye uptake (Fig. 1C, control). Incubation with the Panx-1 blockers such as probenecid (Prob, 500 µM) (Cat# P8761), carbenoxolone (CBX, 50 µM) (Cat# C4790, Sigma Chemical Co. St. Louis, MO, USA), and ^10^Panx peptide (300 µM, PEP) (Cat# 3348, Tocris, Minneapolis, MN, USA) [26,27] did not affect basal Etd uptake observed in PBMCs obtained from uninfected individuals (Fig. 1A, B and C). Further, connexin43 (Cx43) hemichannel blockers such as lanthanum (La^3+^) (Cat# 449,830, Sigma Chemical Co. St. Louis, MO, USA), a general Cx hemichannel blocker, or Cx43^E2^, an extracellular peptide that specifically blocks opening of Cx43 hemichannels [24,28,29] did not have any effect on the low dye uptake observed in uninfected PBMCs (data not shown). No toxic or nonspecific effects of these blockers alone were detected (data not shown). Furthermore, as a control, exposure of PBMCs obtained from uninfected individuals to HIV_ADA_ (20 ng/ml and 0.001 MOI) resulted in a fast and transient opening of the channel as we described [12] indicating that the channels can be open upon the right stimulus.

### 3.3 Pannexin-1 channels are constitutively open on PBMCs isolated from HIV-infected individuals

To assess the opening of the Panx-1 channels on PBMCs isolated from HIV-infected individuals, we used a similar approach as described above. We analyzed Panx-1 channel opening using dye uptake rate on freshly isolated PBMCs from 60 different HIV-infected individuals, mostly with low to undetectable replication, as described in Table 1. All samples analyzed had significant dye uptake, even though most of the individuals did not have the circulated virus at the time of isolation and any sign of extracellular virus was removed by extensive washes (Fig. 1D, E, and data not shown). Our previous data indicated that most opening of Panx-1 channels were transient and were induced by acute exposure to virus [8,12,23]. However, our current data indicate that chronic HIV-infection had profound effects on Panx-1 channel opening via an unknown mechanism that is independent of CD4, CXCR4 or CCR5 engagement for the virus, because soluble CD4, TAK779 or AMD3100 (Cat# SML0911, Cat# A5602, Sigma Chemical Co., St. Louis, MO, USA) did not prevent the spontaneous Panx-1 channel opening observed in PBMCs isolated from HIV-infected individuals (data not shown). As indicated in Fig. 1E, each HIV-infected individual analyzed, had a different time course of Panx-1 channel opening (Fig. 1D–E). The opening of the Panx-1 channels was independent of the age of the individual, gender, years with HIV, CD4 count, HIV replication, ART, and cognitive status.

Panx-1 channel opening on PBMCs isolated from chronic HIV-infected individuals was sensitive to probenecid (Prob, 500 µM), carbenoxolone (CBX, 50 µM), or ^10^Panx peptide (300 µM, PEP) - all Panx-1 channel blockers (Fig. 1F) - indicating that dye uptake was mediated by Panx-1 channels. In contrast, Lanthanum (La^3+^), a general Cx hemichannel blocker, or Cx43^E2^, an peptide that blocks Cx43 hemichannels [24,28,29], did not affect the Etd uptake observed in the PBMCs isolated from our HIV-infected population (data not shown), suggesting that Cx43 hemichannels were not open during our studies. Therefore, we propose that a constitutive opening of Panx-1 channels could explain the chronic inflammation observed in the HIV-infected population, even in the current ART era.

### 3.4 Serum/plasma obtained from HIV-infected individuals had elevated concentrations of inflammatory molecules released upon the opening of pannexin-1 channels

**Fig. 2:**
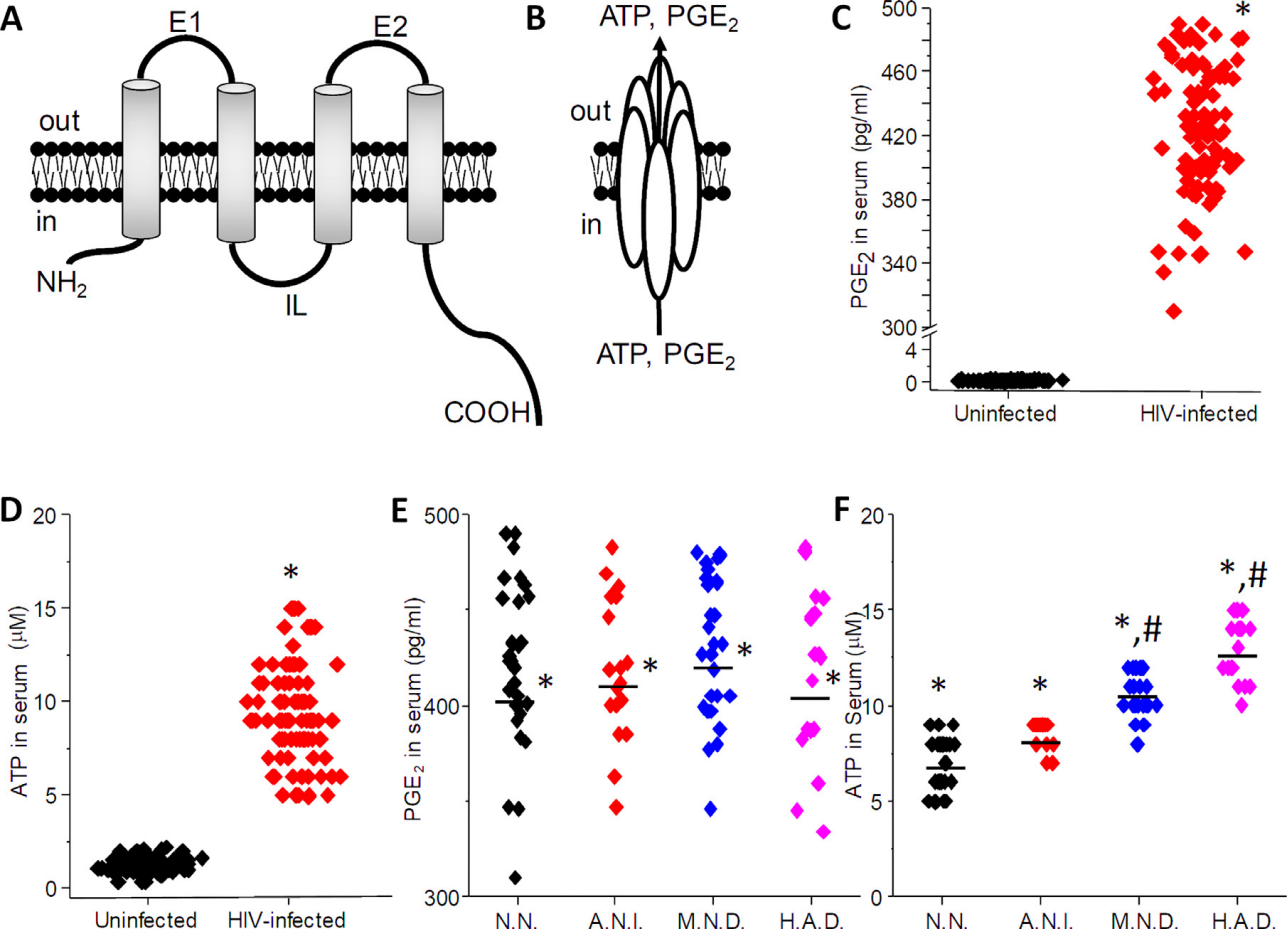
Opening of Panx-1 channels on PBMCs is associated with increased circulating levels of PGE_2_ and ATP in the plasma/serum of HIV-infected individuals. (A) Representation of a Panx-1 protein and the structure of the channel (B). Opening of Panx-1 channels on the surface of the cells enables the release of PGE_2_ and ATP. (C) Quantification of PGE_2_ in serum/plasma by ELISA. Uninfected individuals had low levels of circulating PGE_2_ (black signs). In contrast, serum/plasma obtained from HIV-infected individuals described in Table 1, indicates that all HIV-infected individuals have increased levels of circulating PGE_2_ despite good CD4 counts and low to undetectable viral replication (red signs). (D) Quantification of ATP circulating levels in serum/plasma using ATP light. ATP levels in uninfected individuals were low (*n* = 60). However, in all samples (serum/plasma) obtained from HIV-infected individuals, ATP levels were high (*n* = 115, *p ≤ 0.005 as compared to uninfected conditions). (E) If the PGE_2_ data presented in C were breakdown into cognitive status, N.N.: Neurocognition normal; A.N.I.: Asymptomatic Neurocognitive Impairment; M.N.D.: Mild Neurocognitive Disorder; and H.A.D: HIV-Associated Dementia, no significant differences were observed (*n* = 115, *p ≤ 0.005 as compared to control uninfected conditions). (F) If the ATP circulating levels presented in D were breakdown into cognitive impairment as described above. We identified an association between M.N.D. and H.A.D and the high levels of circulating ATP described in the HIV-infected population. Thus, we propose that circulating ATP levels can be used as a biomarker of cognitive disease in the HIV-infected population (*n* = 115, *p ≤ 0.005 as compared to control uninfected conditions and #*p* ≤ 0.002 as compared to N.N. and A.N.I.). Thus, changes in cognition or CNS compromise are associated with increased levels of ATP. The data are expressed as mean ± SD.

Our published data demonstrated that Panx-1 channel opening in response to HIV-binding to CD4 and CCR5 or CXCR4 enables intracellular ATP to be release to the extracellular space and subsequently activates purinergic receptors, thereby allowing entry of the virus into uninfected macrophages [13]. ATP is released into the extracellular space via three main mechanisms; neuronal secretion (vesicular release), cell death (plasma membrane compromise), and the opening of Panx-1 channels [30], [31], [32], [33], [34] (Fig. 2A and B). Thus, to determine whether the constitutive opening of Panx-1 channels on circulating PBMCs obtained from HIV-infected individuals is also associated with higher levels of intracellular mediators released through the Panx-1 channel, we determined circulating levels of PGE_2_, ATP, TNF-α, and IL-1β in serum/plasma of uninfected and HIV-infected individuals.

As expected, low to undetectable levels of PGE_2_ and ATP were found in the serum/plasma of the uninfected population (Fig. 2C and D, uninfected, respectively, *n* = 60). No detectable levels of TNF-α or IL1β were detected in the serum of uninfected individuals (data not represented). In contrast, high circulating levels of PGE_2_ and ATP were detected in all serum/plasma samples analyzed from HIV-infected individuals (Fig. 2C and D). The differences in circulating levels of PGE_2_ and ATP were independent of the age of the individual, gender, years with HIV, CD4 count, and HIV replication (data not represented). In addition, no detection of TNF-α or IL-1β was found in uninfected samples (data not shown). In conclusion, we demonstrated that intracellular factors such as PGE_2_ and ATP, both released through the opening of Panx-1 channels, are constitutively released into the circulation of all HIV-infected individuals.

### 3.5 Circulating levels of ATP are predictive of cognitive impairment in the HIV-infected population

As described above and by others, under physiological conditions, low concentrations of circulating ATP are found (1–2 µM) [35,36]. However, we found that all HIV-positive individuals analyzed contained significantly higher levels of circulating PGE_2_ and ATP in their plasma/serum relative to uninfected individuals, as described above (*p ≤ 0.005; Fig. 2C and D). PGE_2_ did not associate with cognitive impairment (Fig. 2E). However, when circulating ATP levels were stratified according to cognitive impairment, we found that HIV-infected individuals with no neurocognitive impairments (N.N.) or asymptomatic neurocognitive impairment (A.N.I.) had a significanly lower amount of circulating ATP than individuals with mild neurocognitive disorders (M.N.D.) or HIV-associated dementia (H.A.D.) (*p ≤ 0.005 as compared to uninfected samples; Fig. 2F, ^#^p<0.02 as compared to N.N. and A.N.I. conditions, *n* = 60 for uninfected and *n* = 115 for HIV-infected individuals). We detected that concentrations higher than 8 µM were associated with cognitive disease (M.C.M.D. or H.A.D.) (Fig. 2F). Thus, we propose that circulating levels of ATP could be used as an early biomarker of cognitive impairment in the HIV-infected population.

### 3.6 PBMCs isolated from HIV-infected individuals release ATP and PGE_2_, even without stimulation

**Fig. 3:**
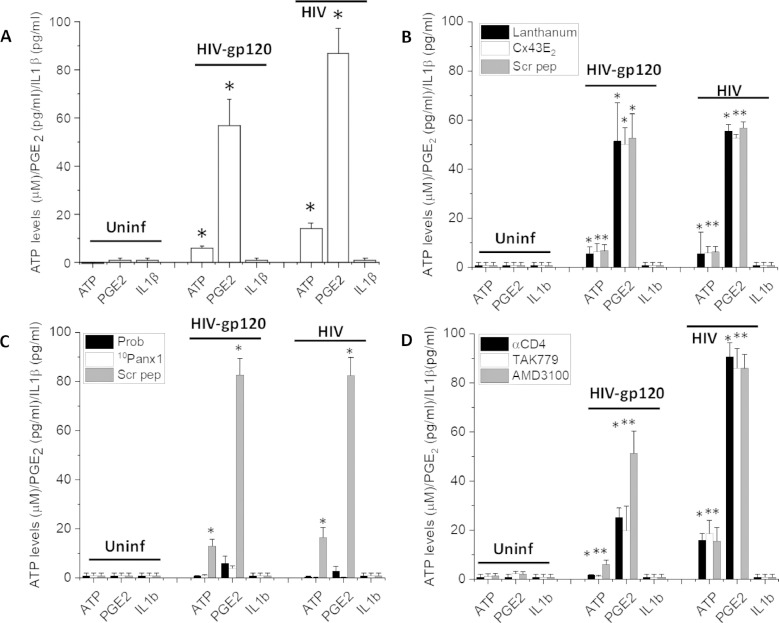
PBMCs isolated from HIV-infected individuals release ATP and PGE_2_ in a Panx-1 dependent, but not Cx43 hemichannel, manner. (A) PBMCs were isolated from uninfected and HIV-infected individuals with and without cognitive impairment, washed, and determinations of ATP, PGE_2_, and IL1β were performed after 15 and 30 min post-stimulation. Uninfected cells (Uninf) PBMCs did not release significant amounts of ATP, PGE_2_, and IL1β. However, upon treatment with the recombinant protein, HIV-gp120 (HIV-Bal), Panx-1 channels become open and a significant amount of ATP and PGE_2_ were released (HIV-gp120). PBMCs obtained from HIV-infected individuals (HIV), without further stimulation, release ATP, and PGE_2_ into the medium. Thus, Panx-1 channels were in an open stage, as described in Fig. 1. In both types of PBMCs, uninfected, and HIV, no detection of IL1β was found. (B) Blocking the opening of Cx43 hemichannels on the surface of PBMCs using lanthanum, or the extracellular peptide (Cx43E_2_) did not alter the release of ATP and PGE_2_ induced by HIV-gp120 or the spontaneous opening observed in HIV infected individuals. Scrambled peptide (Scr pep) did not alter the secretion of ATP and PGE_2_. (C) Blocking the opening of Panx-1 channels on the surface of PBMCs prevented the secretion of ATP and PGE_2_ in uninfected cells treated with HIV-gp120 (HIV-gp120) or in PBMCs obtained from HIV-infected individuals (HIV). (D) The application of soluble CD4 (sCD4) to compete with the binding of HIV-gp120 or the virus prevented secretion of ATP and PGE_2_ in response to HIV-gp120. However, in PBMCs isolated from HIV-infected individuals, no significant effects of sCD4, CCR5 (TAK779) or CXCR4 (AMD3100) blockers were observed. (*n* = 6, *p ≤ 0.003 as compared to control uninfected conditions). The data are expressed as mean ± SD.

To determine whether the opening of Panx-1 channels contributed to the extracellular levels of PGE_2_ and ATP, we determined the acute release of these factors using PBMCs obtained from uninfected and HIV-infected individuals. PBMCs isolated from uninfected individuals did not show the opening of Panx-1 channels (see Fig. 1B), and no significant release of PGE_2_, ATP, and IL-1β into the medium was detected after 15 and 30 min in culture (Fig. 3A, uninf). However, HIV-gp120 treatment (1 µg/ml, from HIV_Bal_) of uninfected PBMCs induced opening of the Panx-1 channels and resulted in the release of ATP and PGE_2_ (5.9 ± 1.3 µM and 56.8 ± 11.1 pg/ml, respectively), but not IL-1β, into the medium after 30 min (Fig. 3A, HIV-gp120). In contrast, PBMCs isolated from HIV-infected individuals washed and placed in culture resulted in ATP and PGE_2_ release into the medium even without any stimulation (14.1 ± 2.4 µM and 87.0 ± 10.1 pg/ml, respectively, Fig. 3A, HIV).

The release of ATP and PGE_2_ in response to HIV-gp120 in PBMCs obtained from uninfected individuals or the spontaneous release observed in PBMCs obtained from HIV-infected individuals was not dependent in the opening of Cx43 hemichannels. Lanthanum, a general Cx hemichannel blocker or an extracellular blocking peptide to the extracellular loop 2 (Fig. 3B, Cx43E_2_) did not affect the release of ATP or PGE_2_ in response to HIV-gp120 using uninfected cells or the release of ATP or PGE_2_ in PBMCs obtained from HIV-infected individuals (Fig. 3B).

In contrast, the release of ATP and PGE_2_ in response to HIV-gp120 in PBMCs obtained from uninfected individuals or the spontaneous release observed in PBMCs obtained from HIV-infected individuals were sensitive to Panx-1 channel blockers (Fig. 3C). Probenecid (Prob, 500 µM) or ^10^Panx1 peptide (300 µM) treatment prevented the release of ATP and PGE_2_ in PBMCs isolated from uninfected and HIV-infected individuals (Fig. 3C). No effects on PGE_2_ and ATP release were detected using the scrambled Panx-1 peptide (Scr pep, Fig. 3C) (Cat# 3708, Tocris, Minneapolis, MN, USA).

Furthermore, blocking gp120 binding to CD4 (soluble CD4 protein, αCD4, Cat. 4615, NIH repository), CCR5 (TAK779, 5 µM) or CXCR4 (AMD3100, 5 µM) also prevented the release of ATP and PGE_2_ in response to gp120 treatment of PBMCs from uninfected individuals (Fig. 3D, HIV-gp120). However, none of the blockers for CD4, CCR5, or CXCR4 reduced the spontaneous opening observed in PBMCs obtained from HIV-infected individuals (Fig. 3D).

Thus, in uninfected PBMCs, HIV stimulation is required to induce the opening of Panx-1 channels as well as to release ATP and PGE_2_. Treatment of uninfected PBMCs with TNF-α (10 ng/ml) (Cat# 11,371,843,001), IL-1β (10 U/ml) (Cat# SRP3083), IFN-γ (10 ng/ml) (Cat# SRP3058) or LPS (1 µg/ml) (Cat# L2630, Sigma Chemical Co., St. Louis, MO, USA) for 30 min does not induce opening of the Panx-1 channels or secretion of PGE_2_ and ATP. Thus, the secretion of these intracellular factors was not associated with immune activation, rather with HIV-infection. In contrast, the release of ATP and PGE_2_ from PBMCs isolated from HIV-infected individuals was independent of CD4 and chemokine receptor stimulation (Fig. 3D). Thus, the mechanism of Panx-1 opening and ATP and PGE_2_ release is not dependent on viral or gp120 binding to host receptors in the HIV-infected population.

In conclusion, chronic HIV-infected PBMCs could be a major contributor to circulating levels of PGE_2_ and ATP observed in the serum of HIV-infected patients. However, we cannot discard another source of circulating PGE_2_ and ATP, such as activated endothelial cells or another cell type.

### 3.7 Opening of pannexin-1 channels is required for enhanced transmigration of HIV-infected lymphocytes and monocytes in response to CCL2

**Fig. 4:**
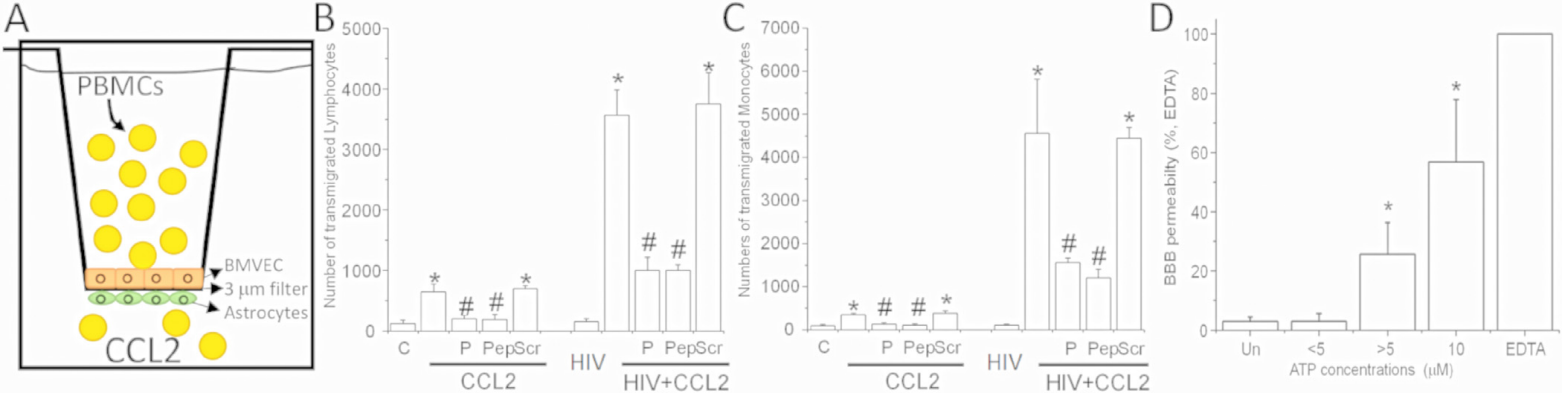
Transmigration of uninfected and HIV-infected PBMCs (HIV_ADA_) in response to CCL2 is Panx-1 dependent and requires secretion of ATP that contributes to BBB disruption often observed in HIV-infected individuals. (A) A schematic representation of our EC/astrocyte blood-brain barrier (BBB) co-culture model. PBMCs (uninfected control and HIV-infected) are added to the top chamber and after 24 h post-transmigration cells in the bottom chamber were collected and stained for CD14 for monocytes and CD3 for lymphocytes. (B and C) Uninfected (C) or HIV-infected (HIV) human PBMCs were added to the top chamber of the BBB model, consisting of co-cultured human ECs and astrocytes in the absence or presence of CCL2 (100 ng/ml) in the bottom chamber to generate a chemotactic gradient. In addition, pre-incubation of PBMCs with probenecid (P) or Panx-1 peptide (Pep) blockers prevented the transmigration of uninfected and HIV-infected lymphocytes and monocytes. Scrambled peptide (Scr) did not alter transmigration of lymphocytes or monocytes in response to CCL2. *p ≤ 0.05 as compared to control conditions without CCL2. # *p* ≤ 0.003 as compared to CCL2 conditions. (D) The permeability of the BBB, without PBMCs, only with added ATP into the top chamber, was determined using albumin conjugated to Evans blue. Untreated BBB model (Un) was impermeable to Evans blue dye. Low levels of ATP detected in uninfected individuals and no cognitive impaired individuals (5 µM) did not result in changes in BBB permeability. ATP concentrations higher than 5 µM (7 and 9 µM) increased the permeability of the barrier. ATP concentrations (10 µM) detected in HIV-infected individuals with M.N.D or H.A.D. compromised BBB permeability. Maximal permeability was reached treating the BBB model with EDTA. *p ≤ 0.005 as compared to untreated conditions. The data are expressed as mean ± SD of 6 experiments.

To determine the role of Panx-1 channel opening in leukocytes, we performed transmigration experiments using a blood-brain barrier (BBB) model that selects HIV-infected PBMCs to transmigrate into the brain side in response to CCL2 [17,22,[37], [38], [39], [40]] (Fig. 4A). This process is a critical hallmark of NeuroHIV.

To determine whether Panx-1 channels, as well as extracellular levels of ATP participate in the transmigration of HIV-infected cells across the BBB in response to CCL2 (100 ng/ml) (Cat# 279-MC, R&D Systems, Minneapolis, MD, USA), we determined transmigration in the presence and absence of Panx-1 channel blockers, probenecid (P) or ^10^Panx-1 blocking peptide (Pep). The addition of uninfected PBMCs to the top chamber of the BBB model in the absence of CCL2 did not affect the baseline permeability of the barrier (data not shown) and minimally affected transmigration of lymphocytes and monocytes across the BBB (Fig. 4B and C, for lymphocytes and monocytes, respectively). Preincubation of PBMCs with Probenecid (P) or ^10^Panx-1 blocking peptide (Pep) prevented transmigration to control levels even in the presence of CCL2 (C, Fig. 4B and C).

PBMCs infected with the HIV_ADA_ were added to the top chamber of BBB co-cultures without CCL2 in the bottom chamber (HIV). After 24 h, there was neither significant transmigration, as described above for uninfected cells (Fig. 4B and C, HIV), nor disruption of BBB impermeability under these conditions (data not shown). The addition of CCL2 to the lower chamber induced high levels of HIV-infected cell transmigration (Fig. 4B and C) and resulted in significant increase in BBB permeability (data not shown), as compared to BBB models after uninfected PBMC transmigration (Fig. 4B and C, bar labeled C on the X-axis). The preincubation of HIV_ADA_ infected PBMCs with Probenecid (P), or ^10^Panx-1 blocking peptide (Pep) prevented transmigration induced by CCL2 and reduced BBB disruption (Fig. 4B and C). Furthermore, the addition of oATP (100 µM, a purinergic receptor blocker) (Cat# A6779) or apyrase (100 units/ml, an enzyme that catalyzes the hydrolysis of ATP) (Cat# A6535, Sigma Chemical Co., St. Louis, MO, USA) to the BBB model blocked transmigration as well as BBB disruption, supporting the hypothesis that opening of Panx-1 channels results in the local ATP release and subsequent activation of purinergic receptors.

### 3.8 High circulating levels of ATP present in HIV-infected individuals compromise BBB permeability

To determine the role of circulating ATP in the HIV-infected population, we measured BBB permeability and leukocyte transmigration across an *in vitro* human BBB model. Both aspects are observed in HIV-infected individuals and several animal models of HIV-brain compromise [40], [41], [42]. In Fig. 4A, we had a representation of the BBB model used to examine permeability and transmigration. Our previous published data indicated that HIV-infection plus CCL2 correspond to a unique combination that favors BBB disruption and enhanced transmigration of HIV-infected leukocytes into the CNS [22,40,43]. However, the mechanism mediating these effects were unknown.

The addition of ATP to the luminal side of the model (blood side) to concentrations lower than 5 µM minimally affected BBB permeability (Fig. 4D, Un). Increasing concentrations of ATP similar to the ones observed in the serum/plasma of the HIV-infected population (higher than 5–10 µM, Fig. 4D), strongly compromised BBB permeability even in the absence of an HIV-component. As a positive control, EDTA (Cat# E6758, Sigma Chemical Co., St. Louis, MO, USA) was used to disrupt the barrier (Fig. 4D, EDTA).

## 4 Discussion

Currently, a major public health problem is the increased prevalence of mild forms of neurocognitive impairment in 50–60% of HIV-infected individuals [44,45]. HIV invades the brain early after primary infection, and despite effective ART, HIV remains in sanctuary sites as viral reservoirs [46], [47], [48]. Although the extent of HIV-infection in the CNS is limited (perivascular macrophages, microglia, and astrocytes), the extent of neuropathogenesis observed does not correlate with viral replication. Thus, it is important to identify biomarkers of CNS disease as well as to understand mechanisms causing CNS compromise.

Currently, CNS damage is evaluated by common neurophysiological testing batteries and by imaging techniques or determination of novel biomarkers in the CSF [49], [50], [51], [52]. However, there are no good systemic biomarkers of HIV-CNS disease. Probably, the most promising CSF biomarker of neuronal injury is neurofilament (NFL) levels that are elevated before the onset of dementia due to neuronal destruction [53], [54], [55]. The normalization of CSF NFL levels is also correlated with the initiation of ART [56], [57], [58], [59]. CSF NFL levels are also elevated in several neuroinflammatory and neurogenerative diseases, as well as stroke and other associated conditions. Thus, NFL CSF levels are not predictive of HIV-associated CNS damage alone, and may just be a late representation of large caliber axon destruction. Furthermore, NFL levels in the periphery are not representative of the damage within the CNS. Thus, the necessity for a peripheral CNS biomarker of disease is urgent.

Various additional potential biomarkers of HIV-CNS disease have been proposed by several groups, including neopterin, BCL11b, beta-2-microglobulin, several markers of inflammation (sCD163, CCL2, TNF-α, IL-6, sCD14, and CXCL10), and interferon-alpha [60], [61], [62], [63]. However, all these biomarkers are associated with already occurring tissue damage and do not predict future damage. Recently, NIH sponsor several groups such as CHARTER, NNTC, Neuroimaging Consortium to collect fluids and tissue speciments. Proteomic determinations of these samples indicated that local alterations in metabolites could predict disease onset. Neuroimaging data also provides essential several potential biomarkers of cognitive disease in the HIV infected population such as N-acetylaspartate (NAA) and creatine (Cr) levels in different regions of the brain; however, the results are contradictory and most of the time are region specific [56,[64], [65], [66]]. Moreover, most of these studies analyzed ratios of these metabolites with respect to Cr, assuming a constant expression of Cr [67], [68], [69]. However, Cr expression is variable with age, trauma, and inflammation. Other key metabolites in the brain are glutamate and glutamine that has been associated with cognitive disease in the HIV-infected population [66]. The combination of these metabolites has become extremely important due to the fact that glutamate and Cr are highly abundant in the brain. Recently has been demonstrated that HIV reservoirs can survive in an alternative source of carbon such as glutamate and glutamine [70]. Additional metabolites and mitochondrial markers are citrate, creatine, glutamine, glucose, inositol, glutamic acid, and CSF mtDNA [62,[71], [72], [73], [74]]. Thus, HIV-infection, even in the absence of replication, has profound effects on the metabolism of infected cells, which may help to perpetuate the virus or promote the survival of viral reservoirs within the brain. This field is just starting, and we believe that our data of ATP dysregulation will contribute to the discovery of new biomarkers of CNS disease in the HIV-infected population that will allow for early intervention before CNS damage becomes irreversible.

Recently our laboratory identified Panx-1 channels, which are membrane-bound large pore channels ubiquitously expressed in all tissues [75], as a key protein and channel involved in HIV pathogenesis [12]. Normally, these channels are in a closed state. However, we identified that binding of the virus to its receptor (CD4) and co-receptors (CXCR4 and/or CCR5) induces the transient opening of Panx-1 channels resulting in ATP release through the channel pore and subsequent purinergic receptor activation to allow HIV entry into immune cells. Pannexins are structurally similar to connexins (Cxs), although they share no sequence homology. Pannexins consist of a cytosolic N-terminal domain, four transmembrane domains with two extracellular loops, and a cytosolic C-terminal domain [76,77]. Currently, there are only a few mechanisms that result in the opening of Panx-1 channels, but most have been described in *in vitro* conditions [76], [77], [78], [79], [80], [81], [82], [83]. Our laboratory found that pathogens, including HIV, can “hijack” these channels to accelerate disease progession [8,[10], [11], [12]]. Thus, the opening of Panx-1 channels is essential for infectivity [12], but any link to NeuroHIV was unknown. Our current data using patient samples indicate that chronic HIV-infection also results in a unspecific opening of Panx-1 channels and the release of several intracellular inflammatory factors into the extracellular space, including ATP and PGE_2_. Normally, secreted ATP is one of the strongest inflammatory signaling molecules in the development of inflammation and tissue regeneration. Given the biological potency of ATP, the control of the duration and magnitude of the cellular responses to ATP is crucial to avoid disease [84,85]. In agreement, its half-life is short and restricted to small areas [86]. Thus, high levels of this nucleotide in the circulation of all HIV-infected individuals analyzed was a surprise and a major finding. Most free ATP is process by ecto-ATPases, which converts ATP to ADP and AMP. For example, the ecto-5′-nucleotidase, CD73, can complete the dephosphorylation process and convert the monophosphate into adenosine. This point is critical because ATP is one of the more powerful pro-inflammatory cytokines, whereas adenosine has a potent immunosuppressive effect [87,88]. Our data, which demonstrate that circulating ATP is more stable in the HIV-infected population, also indicate a significant decrease in adenosine, suggesting that all HIV-infected individuals have problems in removing phosphate from complex structures.

Our study has several limitations that need to be considered. The main mechanism of increased levels of ATP in the HIV-infected population is unknown. Normally, extracellular ATP is degraded quickly, but why remain a significant amount in the blood of HIV infected individuals is unknown. Despite that the number of individuals analyzed was significant (*n* = 175), there was not association of the high levels of ATP present in the HIV-infected individuals with several comorbidities such as alcohol, inflammation, stroke, or infections as well as genetic factors such as ethnicity or gender. However, an increase number of samples could dissect these points. Furthermore, longitudinal studies also could provide a better time line of ATP dysregulation, BBB disruption, and cognitive compromise in the HIV-infected population. A surprising result was that all HIV-infected individuals analyzed have a specific type of inflammation mediated by PGE_2_ and ATP, but not represented in changes in the usual inflammatory factors such as TNF-α or IL1β. Thus, chronic inflammation is present and extremelly specific, despite viral and immune system restauration.

The high and stable ATP concentrations circulating in HIV-infected individuals indicate that Panx-1 channels are open as described in our manuscript, but also indicate an important role of purinergic and adenosine signaling. Our data indicate that ATP and its purinergic receptors are essential for HIV entry, and later stages in viral replication may have potential therapeutic implications. The nearly complete inhibition of viral replication by multiple purinergic receptor antagonists suggests that these receptors may be good targets for therapy. In fact, P2X receptors participate in neuropathic pain, inflammatory disease, and potentially depression, and a number of purinergic receptor antagonists are already in testing for human therapy [89], [90], [91], [92], [93], [94], [95]. Studies using oATP, which we found to be a potent inhibitor of HIV replication and viral entry, in an in vivo mouse model demonstrated that it is a good systemic blocker of purinergic receptors, which can prevent the onset of diabetes and inflammatory bowel disease [96]. Our findings indicate that preventing ATP accumulation or blocking its signaling can reduce the chronic inflammation observed in all HIV-infected individuals and should be considered as potential therapies for HIV-associated comorbidities.

## 5 Role of the funding sources

All NNTC related funding provides a unique human repository for research purposes www.nntc.org. The Alfred P. Sloan Foundation Minority fellowship provides funds to Dr. Velasquez during her Ph.D. training. Funding for J.D.G. provided resources to perform serum analysis in University of North Dakota for ATP, ADP, AMP, and Adenosine. The major funding sources were The National Institute of Mental Health grant, MH096625, the National Institute of Neurological Disorders and Stroke, NS105584, and UTMB internal funding (to E.A.E).The main responsible investigator that has full access to all data and the final responsibility of publication is Dr. Eugenin. None of the funders have any participation in data collection, data analysis, interpretation or writing this report.

## 6 Authors' contributions

S.V., L.P., S.V., A.M.G., and E.A.E. designed the research, analyzed the data, and performed the experiments. M.G., N.K., and J.G. performed a blinded analysis of serum/plasma samples by mass spectrometry to assure proper identification and quantification of the samples. All authors helped to write the manuscript.

## Declaration of Competing Interest

None
